# Quality of intrapartum care at Public Health Institutions of North Achefer District, North West Ethiopia: a mixed method study

**DOI:** 10.1186/s12884-022-04907-5

**Published:** 2022-08-08

**Authors:** Yinebeb Asmare, Tizta Tilahun, Yamrot Debela, Yeshiambew Eshetie, Biniam Minuye, Zemen Mengesha Yalew, Dejen Tsegaye

**Affiliations:** 1 Department of Reproductive Health, College of Health Sciences, Bahir Dar University, Bahir Dar, Ethiopia; 2 Department of Reproductive Health and Population Studies, Bahir Dar University, Bahir Dar, Ethiopia; 3 Department of Health Promotion and Behavioral Science, College of Health Sciences, Bahir Dar University, Bahir Dar, Ethiopia; 4 Department of Nursing, College of Health Sciences, Debre Tabor University, Debre Tabor, Ethiopia; 5 Department of Comprehensive Nursing, College of Medicine and Health Sciences, Wollo University, Dessie, Ethiopia; 6 Department of Nursing, College of Health Sciences, Debre Markos University, Debre Markos, Ethiopia

**Keywords:** Intrapartum care, Quality, Ethiopia

## Abstract

**Background:**

Ending preventable maternal, and neonatal morbidity and mortality cannot be achieved without quality care interventions during the intrapartum and postpartum period. Poor quality care during the intrapartum and postpartum period contributes high burden of maternal and neonatal morbidity. Therefore, the current study aimed to assess the quality of intrapartum care and its associated factors in public health facilities in North Achefer District, North West Ethiopia.

**Method:**

A mixed-type institution-based cross-sectional study design was conducted from November 7 to December 6, 2019. Simple random sampling and purposive sampling were used to select study participants for quantitative and qualitative studies respectively. Data were coded and entered into Epi data version 4.4.2 software and exported to SPSS version 25 for analysis. Variables with a *p*-value of less than and equal to 0.25 were entered into multivariable regression analysis and variables with *p* values < 0.05 were considered statistically significant factors of the quality of intrapartum care. The qualitative data were analyzed by using thematic content analysis. Finally, qualitative findings were used to supplement the quantitative result.

**Result:**

The finding showed that, 27.3% (95% CI: 26.6–28) of mothers received good quality intrapartum care. Presence of long-distance (AOR = 0.19; 95% CI = 0.06, 0.66), health care facility (AOR = 0.07; 95% CI = 0.02, 0.20), and partograph utilization (AOR = 4.9; 95% CI = 1.82, 13.14) were factors associated with the quality of intrapartum care.

**Conclusion:**

The proportion of intrapartum quality care was low. Distance, partograph utilization, and type of health facility were factors associated with quality of intrapartum care. The district, zonal health offices, and regional health bureau should provide capacity building and follow up on partograph utilization, and increase the accessibility of ambulances.

## Introduction

In developing countries, pregnancy and childbirth-related problems continue to be major causes of morbidity and mortality among women of reproductive age [1]. The intrapartum and immediate postnatal periods are a period when both the woman and her child are at risk [2, 3]. In 2017, an estimated 295,000 women died worldwide as a result of pregnancy and childbirth-related causes, with Sub-Saharan Africa accounting for 66% of the global burden. Ethiopia is one of the Sub-Saharan African countries with the highest maternal mortality rate, with an estimated 16,000 maternal fatalities in the same year [4]. Hemorrhage (34%), sepsis/infections (10%), hypertensive disorders (%), and ectopic pregnancy (5%) are the leading causes of mother’s mortality during childbirth [5].

Providing quality health care is important for the best possible medical outcome of mother and neonate through the provision of care that satisfies users and providers; maintaining sound managerial and financial performance [6, 7]. Because the leading causes of maternal death are preventable, initiatives to lower maternal morbidity and mortality focus on facility-based childbirth, skilled attendance at birth, and prompt referral for emergency obstetric treatment if difficulties arise [8, 9], through poor-quality and inaccessible care coexist everywhere in different countries [10].Quality intrapartum care is considered to be low in Sub-Saharan Africa [11, 12], which could be due to a shortage of human resources [13], social, political, and economic factors [14].

Key maternal mortality control strategies include family planning, competent care attendance during prenatal, delivery, and postpartum periods, and emergency obstetric and newborn care have all been recommended in developing countries [15]. Even through maternal health care intervention coverage has increased in many low-income countries over the last decade, poor quality of care remains a challenge [16, 17]. In 2014, the Ethiopian Federal Ministry of Health identified the quality of hospital-based labor and delivery care as crucial priorities for reducing maternal mortality and morbidity [15, 18]. Although, different studies related to maternal health care service utilization, skill birth attendants, place of birth, intrapartum care service utilization [11, 19], are limited. Therefore, the current study was intended to assess the quality of intrapartum care at public health facility of North West Ethiopia.

## Materials and methods

### Study design, period, setting, and population

A mixed type institution-based cross-sectional study was conducted at North Achefer District, North West Ethiopia from November 7 to December 6, 2019. According to 2007 Ethiopia central statistics agency census, the Woreda is located 562 km Northwest of Addis Ababa, 102 km west of Bahir Dar [20] with estimated population of 251,873, of which 22, 9312 living in rural areas [20]. The Woreda has one hospital and seven health centers. There were a round 35 health care workers in the eight health institutions. All women delivered in health care institutions, and the skilled birth attendants of North Achefer district (7 health centers and one primary hospital) were the source population. Women delivered with spontaneous vaginal delivery in health care institutions during the data collection period, and the skilled birth attendants were the study population.

### Sample size determination

The sample size was determined using single proportion formula with an assumption; 95% confidence interval (CI), marginal error (d), 5% and proportion of non-beneficial practice during childbirth in Ethiopia (P),15% [21].

n = [Z a/2]^2 ^* p *q/d^2^= 1.96 * 1.96 * 0.15 * 0.85/0.05 * 0.05=196, with adding 10% for non-response rate; the final sample size was 216 for the quantitative data, and 44 subjects for qualitative data determined through data saturation.

### Sampling technique and procedure

**Fig. 1:**
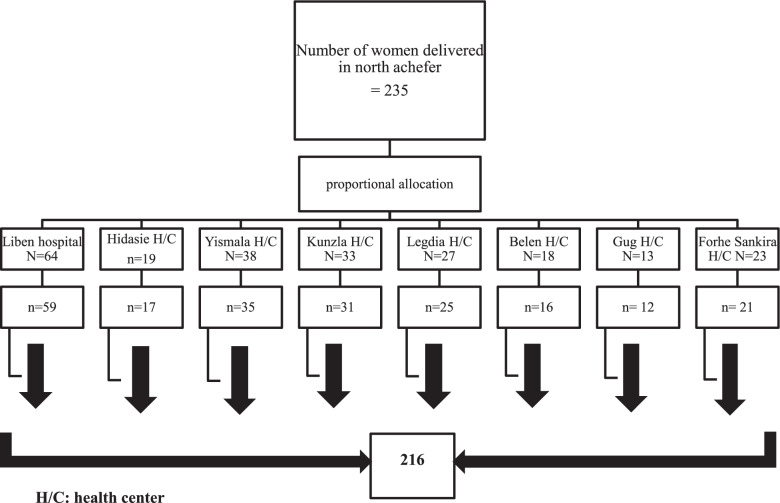
The schematic presentation of participants in North Achefer, 2019

Women delivered in eight health facilities (7 health centers and 1 hospital) from November to December, 2019 were participated in the study. The number of participants from each health care facility was proportionally distributed through taking a one year average number of women who gave birth at each facility and estimating in a month, and then participants from each health institution were selected by simple random sampling technique (Fig. 1). Three focus group discussions with twenty four discussants (skilled birth attendants (health care worker who were currently working in maternity ward)), and with twenty in-depth interviews (women who had secondary and above education, and leaders of women’s health developmental army) were undertaken for the qualitative study until the point in data collection when new insights into the research questions were no longer viable. The participants for qualitative study and.

### Variables

#### Dependent variable

Quality of intrapartum care.

#### Independent variables

##### Socio-demographic related factors

Age of the mothers,, marital status of mothers, number of children’s, and Level of education mothers.

##### Primary care function

Antenatal care follow-up for mothers, Strong emotional support of SBAs and Provider responds politely and respectfully, and use of partograph, skin to skin mother to baby care.

##### Facility level inputs

Facility type the care given, facility opening hour, availability of transportation, Cost for transportation to reach in health facility, and Distance to nearby health facility.

### Operational definitions

Quality is measured using items adapted from the WHO standards similar to the national guidelines [22]

Good quality of intrapartum care: If the individual mothers’ score 75% or more of the intrapartum criteria [11].

Poor quality of intrapartum care: If the individual mothers’ score less than 75% of the intrapartum criteria [11].

### Data collection tool and procedure

The service provision assessment and availability and readiness assessment tool was used. The tool comprises four parts. These are the Sociodemographic characteristics of the mothers (age, sex, educational level, number of children’s and marital status of the mother), primary care function factors (Antenatal care follow-up for mothers, Strong emotional support of SBAs and Provider responds politely and respectfully, and use of partograph, skin to skin mother to baby care), Facility level inputs factors (Facility type the care given, facility opening hour, availability of transportation, Cost for transportation to reach in health facility, and Distance to nearby health facility.), and quality of interapartum care measuring items had five subthemes with yes/no response (quality of health care service measuring items at during admission (21 items), during 1^st^ stage labor(30 items), 2^nd^ stage labor (6 items), 3^rd^ stage labor (10 items), and immediately at postpartum period (23 items)) [11]. Semi-structured observation to observe the mothers and the skilled birth attendants during child birth and immediately at postpartum periods to assess the quality of care and record review form for gathering data from the mothers chart about the completeness of partograph were used to collect quantitative data respectively. In addition, the qualitative data were collected by using an unstructured interviewer guide through focused group discussion and in-depth interview. Eight Bachelor of Science (BSc) Nurses collected the data under the supervision of two Master of Science (MSc) Nurse. An interviewer and a note taker with a recording device were onsite during the interviews.

### Data quality control

Data quality was assured through conducting training for data collectors about the overall process of data collection. The questionnaire was written in English, and then translated into Amharic, which was the study subjects' native language, and finally back to English, by language experts to ensure consistency and conceptual equivalence. At the same time, each completed questionnaire was cheeked for coherence, completeness, and consistency. The daily evaluation was carried out to address any issues that arose during the data collection process. Field notes and audio recordings were examined for appropriateness in terms of accurate coding and audibility for qualitative data. Furthermore, transcriptions were completed in a quiet environment.

### Data processing and analysis

The overall quality care was determined through the summation of the quality of intrapartum care measuring items. After data analysis, descriptive statistics such as proportions, percentages, frequency distribution, and graphical presentation were used to describe the data. Variables with a *p*-value of less than and equal to 0.25 were entered into multivariable regression analysis and variables with *p* values < 0.05 were considered as statistically significant factors of quality of intrapartum care. For qualitative data, verbatim transcription in the Amharic language was made. The transcribed text file was translated into the English language for analysis. The translated text file was analyzed by using thematic content analysis. Finally, qualitative findings were used to supplement the quantitative result.

## Result

### Socio-demographic, primary care function, and facility level inputs characteristics of mothers

A total of 216 mothers delivered in 8 health facilities involved in the quantitative study. The mean age of the mothers was 28.4 (SD ± 4.9) years. Two hundred nine, (96.8%) mothers were married, and 124 (57.4%) had children less or equal to two years. Ninety-five mothers, 44% did not pay for transportation to reach the health facilities, (Table 1).

**Table 1:** Socio-demographic characteristics of mothers delivered in North Achefer health care facilities, North West Ethiopia, 2019

Variables	Frequency No	Percentage %
Age		
18–23 years	34	15.7
24–29 years	90	41.7
30–35 years	76	35.2
≥ 36 years	16	7.4
Educational status		
Unable to read and write	121	56
Able to read and write	30	13.9
Primary education	24	11.1
Secondary education	21	9.7
Diploma and above	20	9.3
Number of children		
≤ 2	124	57.4
3–5	77	35.6
≥ 6	15	7.0
Cost for transportation to reach in health facility		
Not at all affordable	33	15.3
Moderately affordable	29	13.4
Affordable	59	27.3
Free fee	95	44
ANC follow-up with husband		
No	95	44
Yes	121	56
Type of health facility the care given		
Health center	157	72.7
Hospital	59	27.3
Respond to mothers politely an d respectfully		
Yes	164	76
No	52	24
Skin to skin mother—baby care		
Yes	13	6
No	203	94
Facility opening hour		
Not at all suitable	26	12
Moderately suitable	31	14.4
Suitable	159	73.6
Transportation availability		
Difficult	62	28.7
Moderately difficult	38	17.6
Not at all difficult	116	53.7
Distance from the health facility		
Long	66	30.6
Quit long	54	25
Short	96	44.4
Use of partograph to follow the progress of labor		
Yes	130	60.2
No	86	39.8

### Quality of intrapartum care

**Fig. 2:**
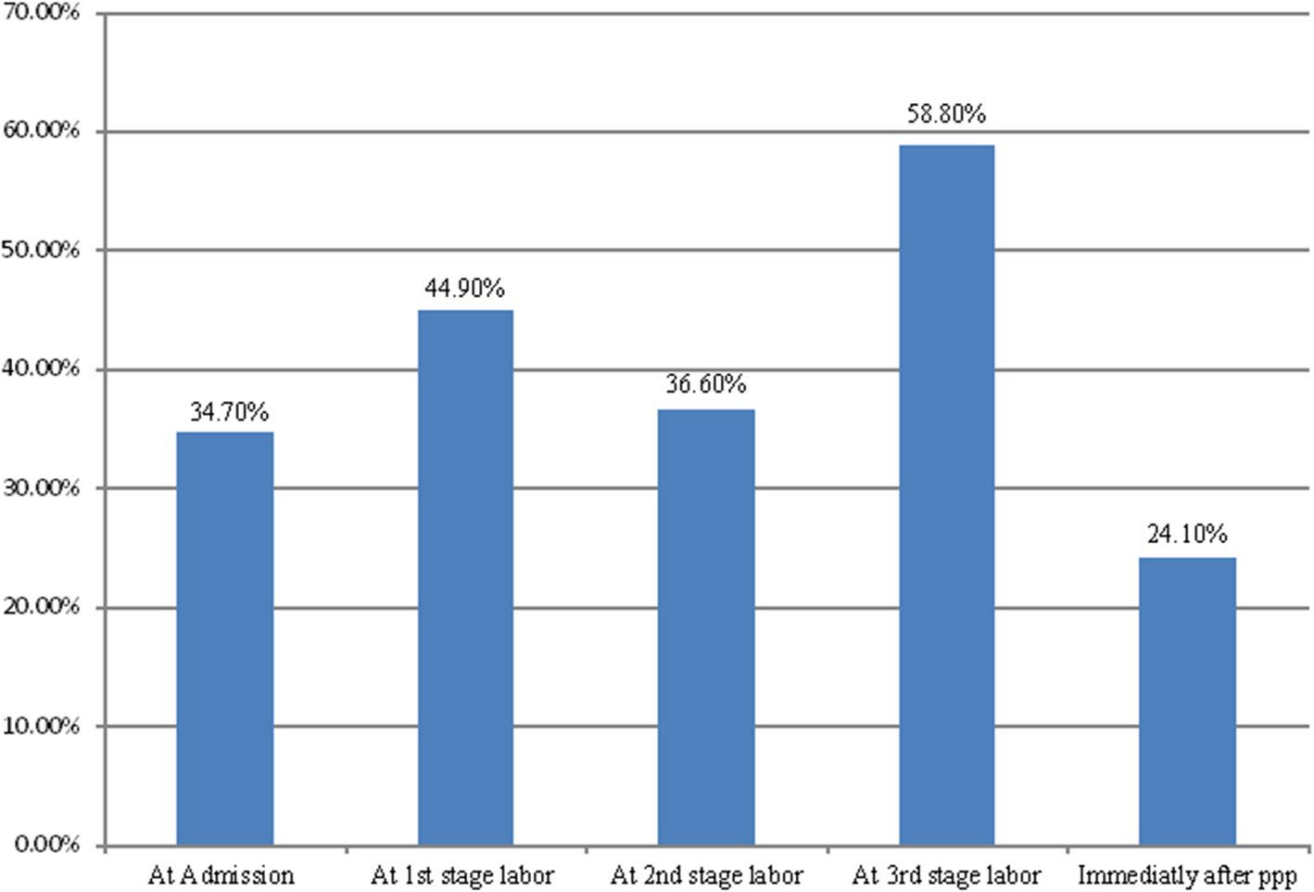
Quality of intrapartum care from admission to immediately at postpartum period given to mothers in North Achefer, 2019

A total of 216 women and 44 skilled birth attendants, and women’s for quantitative and qualitative studies were involved respectively. After sum up the intrapartum measuring items (health care quality measuring items under subthemes such as health care given during admission, at 1^st^ stage labor, 2^nd^ stage labor, 3^rd^ stage labor, and immediately at postpartum period), 27.3% (95% CI: 26.6–28) of mothers were received good quality intrapartum care (Fig. 2). This was supported with qualitative findings. In the qualitative study 2 main themes (quality of intrapartum care and challenges or factors for quality of intrapartum care) were created. Majority of the discussants and the interviewee share the idea that the health care delivery services that they gave to mothers during delivery were poor. One of the discussants from the health center said that;

*“Many mothers nowadays give birth in hospitals, even if their prior pregnancy was delivered at home. Although some mothers received some basic services, the majority of mothers received unsatisfactory health care. In general, the quality of care provided to mothers in this health center falls far short of what is expected’’***(28 years male midwife).**

One of the interviewed mothers from the health center said that

*“…. I was dissatisfied and unsure about the appropriateness of the service provided to me and my newborn since they were inattentive and lacked confidence” ***(Para 3, 32 years old mother).**

Another male discussant added on this position:

*‘‘….mostly we professionals are not updating ourselves with different guidelines, and the care which is given in this health center we can say it is not based on the standard. Therefore the hospital should mentor and correct the health centers. Health care providers at health centers and hospitals should receive various trainings so that they can improve their working processes. If such is the case, I believe mothers will receive better treatment"***(24 years old midwife).**

## Factors associated with good quality of intrapartum care

Long distance, Type of health facility the care given (health center), and partograph utilization were factors associated with good quality intrapartum care.

The odds of mothers who came from a long distance from health facilities were 81% less likely to get a good quality intrapartum care than those who come a shorter distance from health facilities (AOR = 0.19, 95% CI: 0.06, 0.66). This was supported by qualitative finding. A 25 years old male said:

*‘‘The first problem I see in providing appropriate care to mothers is distance, especially in rural and remote places where neither an ambulance nor a motorcycle can reach them; as a result, mothers confront a challenge in receiving care” ***(health officer).**

A 45 para 3 mother added:

*“Because of the distance, getting health care from health facilities has been difficult. We are from a distant rural area where we are unable to obtain adequate health care; therefore we have chosen to remain at home”.*

Women who gave birth at health center were 93% less likely to get good quality intrapartum care than those delivered at the hospital (AOR = 0.07, 95% CI: 0.02, 0.20). In addition, those mother whose progress of labor were followed using partograph were 4.9 times more likely to receive a good quality of intrapartum care than their counterparts (AOR = 4.9, 95% CI: 1.82, 13.14) (Table 2). A 34 years male suggested:

*“…mostly in our health center, we professionals do not use partograph during labor; the main reason is that most health workers do not have basic skill to use it”***(Nurse).**

**Table 2:** Factors associated with good quality of intrapartum care in public health facilities of North Achefer district, 2019

	Quality Intrapartum Care				
Variables	Good No. (%)	Poor No. (%)	COR (95%CI)		AOR (95%CI)
Distance from health facility					
Long	17 (25.8%)	49 (74.2%)	0.20 (0.08, 0.54) **		0.19 (0.06, 0.66) *
Quite long	6 (11.1%)	48 (88.9%)	0.58 (0.29, 1.15)		0.40 (0.12,1.11)
Short	36 (37.5%)	60 (62.5%)	1		1
Use of partograph to follow the progress of labor					
Yes	51 (39.2%)	79 (60.8%)	6.29 (2.81, 14.13) **		4.90 (1.82, 13.14) *
No	8 (9.3)	78 (90.7)	1		1
Type of health facility care given					
Health center	22 (14%)	135 (86%)	0.10 (0.05, 0.19) **		0.07 (0.02, 0.20) **
Hospital	37 (62.7%)	22 (37.3%)	1		1
Mother came ANC follow-up with husband					
Yes	42 (34.7%)	79 (65.3%)	2.44 (1.28, 4.65)		1.13 (0.47, 2.69)
No	17 (17.9%)	78 (82.1%)	1		1
Respond to mother politely and respectful manner					
Yes	52 (31.7%)	112 (68.3%)	2.99 (1.26, 7.06)		1.69 (0.57, 5.03)
No	7 (13.5%)	45 (86.5%)	1		1
Skin to skin mother-baby care					
Yes	5 (38.5%)	8(61.5%)	1.72 (1.05, 4.23)		1.13 (0.26, 2.83)
No	54 (26.6%)	149 (73.4%)	1		1
Facility’s opening hours					
Not at all suitable	3 (11.5%)	23 (88.5%)	0.45 (0.15, 0.85)		0.23 (0.13, 1.12)
Moderately suitable	7 (22.6%)	24 (77.4%)	0.65 (0.44, 11.52)		0.31 (0.79, 7.21)
Suitable	49 (30.8%)	110 (69.2%)	1		1
Transport condition					
Difficult	6 (9.7%)	56 (90.3%)	0.23 (0.2, 0.87)		0.14 (0.29, 2.79)
Moderately difficult	12 (31.6%)	26 (68.4%)	0.84 (0.08, 0.53)		0.48 (0.18, 3.36)
Not at all difficult	41(35.3%)	75 (64.7%)	1		1
Educational status of the mother					
Unable to read & write	22 (18.2%)	99 (81.8%)		0.44 (0.07, 0.98)	0.21 (0.73, 2.31)
Able to read & write	10 (33.3%)	20 (66.7%)		0.83 (0.12, 1.4)	0.46 (0.8, 2.45)
Primary education	9 (37.5%0	25 (62.5%)		0.87 (0.1, 1.1)	0.50 (0.5, 2.13)
Secondary education	6 (28.6)	15 (71.4%)		0.90 (0.05, 0.41)	0.53 (0.47, 2.69)
Diploma and above	12 (60%)	8 (40%)		1	1

^*^Significant at *p*- value < 0.05

**significant at *p*- value < 0.001

A 24 male added:

*‘‘We asked the administrative bodies to increase duty staff for improving the quality of care, but still no response. Since one person was in duty, we face difficulty in utilizing the patrograph appropriately, so quality affected’’***(Midwife).**

## Discussion

The proportion of good quality intrapartum care was 27.3% (95% CI: 26.6–28). Correspondingly, the qualitative finding showed that nearly all respondents mentioned, health care delivery services that they gave to mothers during delivery were poor. The finding was consistent with a study in Tigray region,29.2% [11]. However, lower than a study done in India, 89.7% [23]. This possible reason could be due to the study in India was carried out for eight months duration with coaches; whereas the current study is for only one month. The recent finding was higher than a study in Tanzania, 14% [24]. The difference might be due to differences in quality assessment method, sample size, and setting. The study conducted in Tanzania covered large catchment area with community based survey, and the assessment method focused of the direct response of mothers with five point likert responses.

In this study, mothers who live a long distance from health facilities were less likely to get a good quality of intrapartum care than those who live a short distance from health facilities. Similarly the qualitative finding supports the quantitative finding. This finding also consistent with studies done in Ghana [25], and low and middle-income countries [26, 27]. The reason could be related to lack of dedicated transportation to reach health facility, especially at night.

Mothers whose labor progress was followed using partograph by health care providers were more likely to receive a good quality of intrapartum care than their counter parts. This finding was in accordance with studies conducted in the Tigray region, Ethiopia [11], Ethiopia’s Hospitals [28], and Thai-Myanmar [29]. This showed a lack of attitude, skill, and knowledge among service providers about partograph completion was reported similarly in Ethiopia. Partograph misuse or non-use can delay treatment in cases of prolonged labor, which can result in complications such as obstructed labor, ruptured uterus, bleeding, fetal death, and infections of both mothers and babies [21].This means that partograph utilization is one of the indicators of progress of labor, which in turn increase the quality of care.

The present study also showed that mothers who delivered at the health center were less likely to get a good quality intrapartum care than those who delivered at the hospital. This finding agrees with the studies conducted in low and middle-income countries [30], and Korea [31]. The possible reasons may be hospitals were mainly found in the town with better access to skilled human resources, frequent supervision, better resource, and referral linkage.

### Limitation of the study

Social desirability bias could be the limitation of this study. Respondents were assured complete anonymity and confidentiality throughout the study, which is important to reduce social desirability bias.

## Conclusion

In this study, the quality of intrapartum care was below the standard. Distance from mothers’ homes to the nearby health facility, type of health facility care given, and use of partograph to follow the progress of labor were factors associated with the quality of intrapartum care. Therefore, the district, zonal and regional health bureau should work to increase the accessibility of ambulances quickly to remote areas, and conducting on job training sessions on partograph utilization. We recommend researchers conduct follow-up studies that focus quality care on maternal and neonatal outcomes.

## Data Availability

All relevant data are within the manuscript.

## Abbreviations

AOR: Adjusted Odds Ratio
COR: Crude Odds Ratio
FGD: Focus Group Discussion
FMOH: Federal Ministry of Health
WHO: World Health Organization
